# Bilateral orbito-cerebral-extending frontal mucocele following nasosinus polyposis: A case report

**DOI:** 10.1016/j.amsu.2021.102432

**Published:** 2021-05-28

**Authors:** Khalid Bouhafs, Azeddine Lachkar, Tayeb Bouamama, Drissia Benfadil, Mohammed Rachid Ghailan

**Affiliations:** a Otorhinolaryngology and Head and Neck Surgery Department, Mohammed VI University Hospital, Oujda, Morocco; b Faculty of Medicine and Pharmacy, Mohammed 1st University, Oujda, Morocco; c Department of Radiology, Mohammed VI University Hospital, Oujda, Morocco

**Keywords:** Frontal mucocele, Orbital-cerebral extension, External route, SCARE guidelines

## Abstract

**Introduction:**

and importance: Mucoceles are expansive pseudocystic formations, developed from the sinuses of the face, affecting mainly adults. Evolving at low noise, they are most often revealed by neurological or ophthalmological complications. We report a rare case of a bilateral frontal mucocele with orbito-cerebral extension following nasal sinus polyposis.

**Case presentation:**

This was a 35-year-old patient with a history of Widal syndrome, who presented frontal headaches and left proptosis evolving for 4 months, in whom clinical examination revealed a left superomedial eyelid swelling, left proptosis and stage 2 nasosinus polyposis. Computed tomography and craniofacial magnetic resonance imaging were in favor of a bilateral frontal mucocele with left orbital and bilateral cerebral extensions. The patient was bilaterally operated by a combined approach including external Jacques eyebrow and endonasal Draf IIa procedure in addition to a radical total ethmoidectomy. The outcomes were favorable with regression of headaches and resolution of exophthalmos.

**Clinical discussion:**

The frontal mucocele, although benign, has an aggressive potential in the absence of treatment either towards the endocranium or the orbit behind the orbital septum causing intra-orbital extension, or in front of it; causing the dominant upper palpebral form as in the case of our patient. The treatment is still based on surgical excision of the cyst with drainage of the causal sinus, which was carried out for our patient.

**Conclusion:**

Despite its benign behavior, frontal mucocele may become serious by compression of neighboring organs which require an early and appropriate surgical management.

## Highlights

• Mucoceles are rare benign sinus tumors of the frontoethmoid complex.
• They rarely extend to the endocranial anatomical structures.
• Imaging is of capital importance for its multidisciplinary management.
• Surgery is the standard treatment of mucocele based on external and endonasal routes.

## 1 Introduction

Mucoceles are expansive cystic pseudotumors of the paranasal sinuses limited by a respiratory epithelium and filled with mucus, following the obstruction of the causal sinus drainage pathways [1]. Hence, their progression is very low and often asymptomatic. Therefore, this is associated with a diagnostic delay as they are only discovered at the stages of serious orbital or endocranial complications [2,3]. This rare tumor involves frequently the frontoethmoid complex [1] and it rarely extends to the endocranial anatomical components [4]. In this paper, we report a case of bilateral frontal mucocele with orbito-cerebral extension following nasosinus polyposis and we discuss this presentation based on a review of the literature. The current case is reported as recommended by SCARE guidelines [5].

## 2 Case presentation

**Fig. 1:**
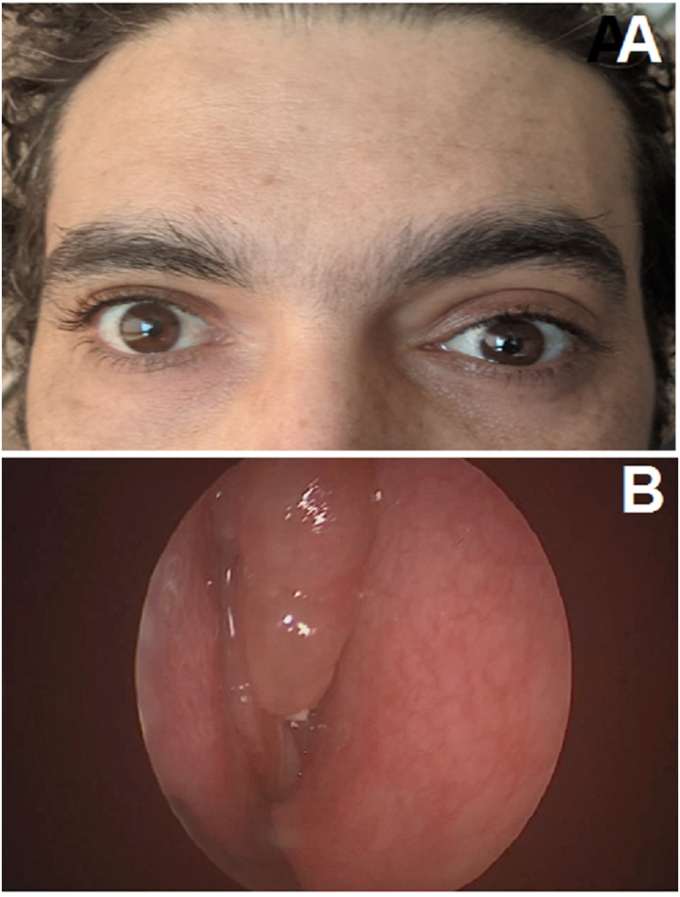
**(a)** Clinical appearance of a left frontal mucocele and **(b)** Endoscopic appearance of a stage 2 nasosinus polyposis.

**Fig. 2:**
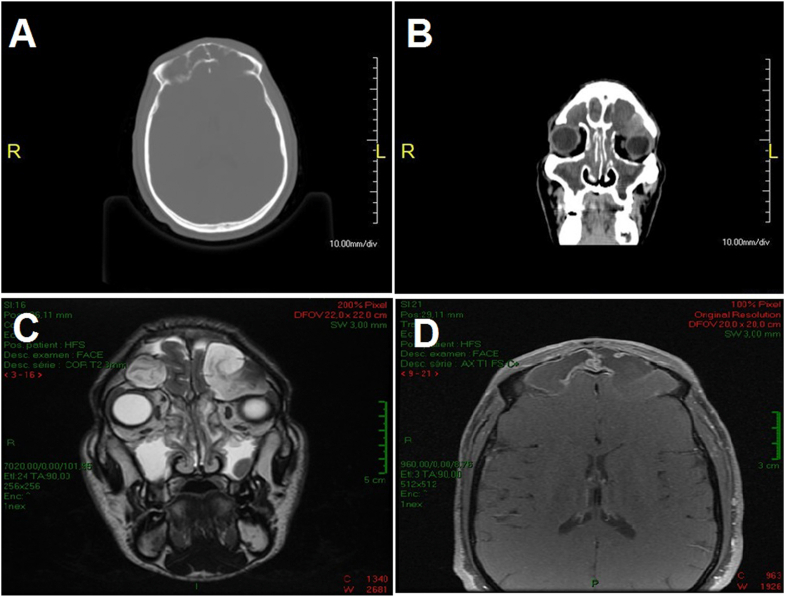
**(a)** CT bone window in axial section showing total filling of the frontal sinuses with blown appearance of the sinus walls, **(b)** CT parenchymal window in coronal slice showing 2 frontal mucoceles with left orbital extension, **(c)** Coronal T2 weighted MRI showing an expansive process of the frontal sinuses with hyperintensity lowering the roof of the left orbit, and **(d)** Axial T1-weighted MRI after injection showing a hypointense lesion of the frontal sinuses, enhancing peripherally with bilateral endocranial extension.

Our patient was a 35-year-old male with a history of Widal syndrome who presented frontal headache, resistant to the usual analgesics associated with a left proptosis that progressed for 4 months. The ophthalmological examination found a left upper-medial eyelid swelling, which was renitent, non-beating, without inflammatory signs. A left non-axile, irreducible, painless, and impulsatile exophthalmos without a loss of visual acuity or ocular motricity was noticed in our patient (Fig. 1A–B). Furthermore, the otorhinolaryngological examination found a stage 2 nasosinus polyposis (Fig. 1B) and the neurological examination was unremarkable. On imaging, craniofacial computed tomography (CT) showed a total sinus filling of the face and two hypodense formations centered on the frontal sinuses, with thin walls which were not enhanced after injection of contrast. These formations were blowing and eroding the sinus bone walls, extending into the intra-cranial area with a compression of the two frontal lobes and intra-orbital left associated with grade 2 proptosis and measuring 40 × 25 mm and 30 × 23 on the left and the right side respectively (Fig. 2A and B). Cranio-facial magnetic resonance imaging (MRI) revealed an expansive process of the frontal sinuses, laminating the sinus bone walls which was hypointense in T1 and heterogeneous in T2 hypersignal and enhancing at the periphery after injection of contrast product (41 × 33 mm on the left and 29 × 33 mm on the right). This lesion lowered the roof of the left orbit and the ipsilateral superior right muscle with bone lysis, with a marked grade 2 exophthalmos and bulging into the right orbital and cranial cavities by compressing the anterior pole of the frontal lobe (Fig. 2C and D). Several diagnoses were proposed including epidermoid cysts, cholesteatomas, meningiomas, chordomas, encephaloceles and mucoceles, neurofibromas, and other tumors. MRI-based imaging was decisive in considering mucocele as the diagnosis that can explain the patient clinical presentation.

**Fig. 3:**
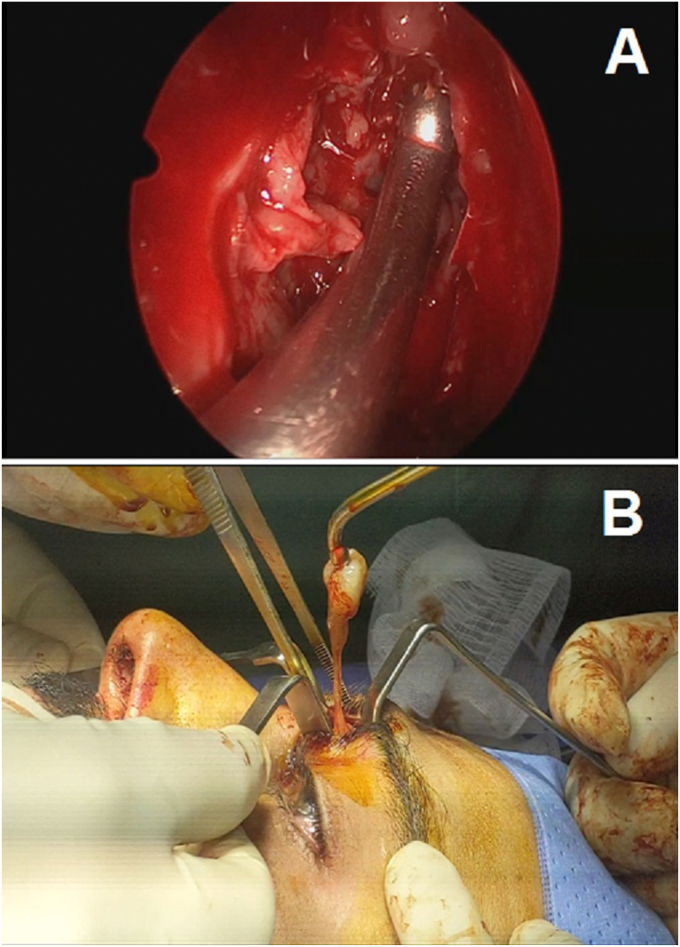
**(a)** Frontal sinusotomy through Draf IIa and **(b)** Exeresis of the frontal mucocele through the eyebrow approach of Jacques.

Our patient benefited from a surgical removal using a bilateral approach and he initially underwent an endoscopic permeabilization surgery of the frontonasal drainage tract through a Draf IIa procedure in addition to a radical total ethmoidectomy (Fig. 3A). In view of the laterality and the extension of the mucocele, the excision of the mucocelic membrane based on Jacques's eyebrow approach enlarged to the outer part of the eyebrow (Fig. 3B). Surgery was performed by a senior professor of oto-rhino-laryngology assisted by resident trainees in the same specialty. The histological examination confirmed our diagnosis. With a follow-up of 3 months, the evolution was favorable with good healing of the operative wound, regression of headaches and resolution of the exophthalmos.

## 3 Discussion

Mucoceles are rare benign sinus tumors and in 90% of cases affect the frontoethmoid complex without gender difference [[6], [7], [8], [9], [10]]. Our patient, with an age of 35, is younger compared to what has been reported previously [[11], [12], [13], [14]]. The origin of mucoceles is variable and occurs mainly in adulthood, a few rare pediatric cases of mucoceles have however been reported [7,[13], [14], [15]]. Regarding its pathogenesis, it generally results from an obstruction of a sinus ostium and an inflammatory mucosal reaction. According to Molteni and al. [16], the obstruction of the nasofrontal canal by an inflammatory scar process (sinusitis or nasal sinus polyposis) as testified by our patient, traumatic, surgical or tumor is incriminated in 64% of the reported cases. Among the traumatic factors, iatrogenic surgery on the nasofrontal canal has been reported by several authors [6,8,10]. In the remaining 36%, the etiology remains undetermined.

Mucoceles classically evolve in two evolutionary phases [15] encompassing an initial asymptomatic phase and an exteriorization or complication phase with clinical expression. The clinical signs that may reveal this entity are ophthalmologic, rhinologic or neurologic. Therefore, the clinical picture associating a swelling of the frontal seat or at the level of the supero-internal angle of the orbital frame with an exophthalmos is characteristic of the frontal mucocele as in our case for the left frontal mucocele while that right was incidentally discovered on imaging. The development of these lesions is long and insidious varying between 2 months and 25 years [12]. In our case, this period was 4 months. Given its aggressive potential, a frontal mucocele can spread in the absence of treatment either towards the endocranial cavity by erosion of the posterior cortical wall of the frontal sinus which is the most serious but rare evolution and can led to meningoencephalic complications or rhinorrhea [4]. This entity can also extend to the orbital cavity by erosion of the anterior cortical wall which can be the origin of a lateralized exophthalmos. Moreover, these two locations can also be associated as in the case reported in this paper. Thus, headaches observed in our patient are dueto the intracranial development of the mucocele exerting a mass effect on the frontal lobe. The mode of oculo-orbital extension of the frontal mucocele depends on the site of the anterior opening of the frontal sinus in relation to the orbital septum. If the opening is located in the front area, the frontal mucocele will extend further in the thickness of the upper eyelid like our case. On the other hand, if this opening occurs behind, the extension of the frontal mucocele will be inside the orbit. These two types of oculo-orbital mode of frontal mucocele extension can influence the treatment decisions and the expected outcomes. The craniofacial CT scan is now the firstline imaging procedure to diagnose the mucocele. It can also assess the associated bone lesions such as osteomas of the paranasal sinuses, craniofacial fibrous dysplasia and/or complications including abscess, intra-orbital rupture, bone lysis, and brain involvement. According to the classification proposed by Thiagarajan [17], our patient was categorized as type Va before erosion of the anterior and posterior walls of the sinus with minimal intracranial development. MRI, with better resolution of tissue contrast, is considered as the gold standard as it better specifies the extension to the adjacent structures [13,18] and also allows the exclusion of differential diagnoses such as encephaloceles, epidermoid cysts, cholesteatomas, meningiomas, chordomas, neurofibromas, and polyploid tumors. The imaging workup is also of capital importance in the choice of the approach, and therefore for the multidisciplinary team involved in the treatment [19]. Surgery is the standard treatment of mucocele based on two approaches including external and endonasal routes or also via an endoscopic endonasal strategy. Several surgical techniques can be used for this purpose mainly those using gingivo-jugale route of Caldwell-Luc for maxillary mucoceles and the eyebrow route of Jacques for frontal or fronto-ethmoidal mucoceles as well as the bicoronal approach of Cairne Unterberger [16].

The treatment is based on the reintegration of the sinus housing the mucocele into the respiratory system (nasalization) or its exclusion by filling or cranialization. The principle of the external pathway is similar and is based on the large communication of the sinuses with the normal drainage system [20]. Endoscopic surgery by endonasal route is currently accepted as the method of choice in the management of mucoceles due to its low iatrogenicity and its excellent efficiency. This approach enables a respect of the healthy mucosa and a widening the natural drainage routes [21,22]. However, it should be indicated for lateral and extensive frontal localizations, skin fistulizations, and suspicion of associated malignant lesions and for the prevention of the risk of recurrence in the event of associated inflammatory ethmoidal pathology [23]. Our patient was operated bilaterally by a combined route using an endonasal approach by frontal sinusotomy through a Draf IIa in addition to a radical total ethmoidectomy given the failure of the well-conducted medical treatment of the nasosinus polyposis and also by using external approach. Long-term monitoring should be provided, as nasosinus mucoceles can recur several years after the initial surgery at a rate varying from 3 to 35% [24]. The 3-month follow-up of our patient is insufficient to judge the long-term evolution of the mucocele. However, the prognostic outcomes in these cases are most often good, with a resolution of the exophthalmos and a regression of headaches. The patient was satisfied with our management.

## 4 Conclusion

Frontal mucoceles are benign lesions. Their severity is due to their aggressive potential towards neighboring organs which may be associated with functional or even vital prognosis. Their management was improved with the advent of imaging and endonasal surgery which is considered as the gold standard. In our setting, external surgery seems desirable as these cases are diagnosed at advanced stages with endocranial expansion.

## Consent

Written informed consent was obtained from the patient for publication of this case report and accompanying images. A copy of the written consent is available for review by the Editor-in-Chief of this journal on request.

## Ethical approval

Not required.

Provenance and peer review.

Not commissioned, externally peer-reviewed.

## Sources of funding

None.

## Author contribution

Dr Khalid Bouhafs: writing the manuscript, Review and Editing. Professor Azeddine Lachkar: Visualisation, Conceptualisation and Investigation. Dr Tayeb Bouamama: provided the imaging data of the patient. Professors Drissia Benfadil and Mohammed Rachid Ghailan supervised the writing of manuscript. All authors also declare that they have read and approved the final version of the manuscript.

## Research registration

N/a.

## Guarantor

The Guarantor is the one or more people who accept full responsibility for the work and/or the conduct of the study, had access to the data, and controlled the decision to publish.

## Declaration of competing interest

The authors have no conflict of interest to declare.
